# Exhaled nitric oxide is related to atopy, but not asthma in adolescents with bronchiolitis in infancy

**DOI:** 10.1186/1471-2466-13-66

**Published:** 2013-11-17

**Authors:** Ingvild Bruun Mikalsen, Thomas Halvorsen, Knut Øymar

**Affiliations:** 1 Department of Paediatrics, Stavanger University Hospital, Stavanger, Norway; 2 Department of Clinical Science, University of Bergen, Bergen, Norway; 3 Department of Paediatrics, Haukeland University Hospital, Bergen, Norway

**Keywords:** Children, Eosinophilic inflammation, Respiratory syncytial virus, Wheezing

## Abstract

**Background:**

The fraction of exhaled nitric oxide (FeNO) has been suggested as a non-invasive marker of eosinophilic inflammation in asthma, but lately rather as a biomarker of atopy than of asthma itself. Asthma after bronchiolitis is common up to early adolescence, but the inflammation and pathophysiology may differ from other phenotypes of childhood asthma. We aimed to assess if FeNO was different in children with former hospitalization for bronchiolitis and a control group, and to explore whether the role of FeNO as a marker of asthma, atopy or bronchial hyperresponsiveness (BHR) differed between these two groups of children.

**Methods:**

The study included 108 of 131 children (82%) hospitalized for bronchiolitis in 1997–98, of whom 82 (76%) had tested positive for Respiratory syncytial virus, and 90 age matched controls. The follow-up took place in 2008–2009 at 11 years of age. The children answered an ISAAC questionnaire regarding respiratory symptoms and skin prick tests, spirometry, methacholine provocation test and measurement of FeNO were performed.

**Results:**

Analysed by ANOVA, FeNO levels did not differ between the post-bronchiolitis and control groups (p = 0.214). By multivariate regression analyses, atopy, height (p < 0.001 for both) and BHR (p = 0.034), but not asthma (p = 0.805) or hospitalization for bronchiolitis (p = 0.359), were associated with FeNO in the post-bronchiolitis and control groups. The associations for atopy and BHR were similar in the post-bronchiolitis and in the control group.

**Conclusion:**

FeNO did not differ between 11 year old children hospitalized for bronchiolitis and a control group. FeNO was associated with atopy, but not with asthma in both groups.

## Background

Asthma in childhood is characterized by extensive heterogeneity regarding aetiology and natural history, and may present with various phenotypes probably related to different immunological, inflammatory and airway characteristics [1]. Chronic inflammation of the lower airways and bronchial hyperresponsiveness (BHR) are typical features of asthma, and markers of these factors are therefore used for diagnostic purposes and to guide treatment. The fraction of exhaled nitric oxide (FeNO) has been suggested as a non-invasive marker of eosinophilic inflammation [2], and thus a marker of asthmatic airway inflammation. Recently, FeNO has been suggested as a biomarker of atopy, and thereby a biomarker of atopic asthma rather than of asthma per se [3-5], although findings have been equivocal [6-8]. Associations between FeNO and BHR, but not between FeNO and asthma have been described in atopic children [5].

Bronchiolitis in early life is an established risk factor for subsequent asthma, although the mechanisms behind are complex and heterogeneous [9]. The risk of asthma is higher after RSV negative than RSV positive bronchiolitis [10], particularly after early wheezing or bronchiolitis due to Rhinovirus (RV) [11]. While atopic asthma is associated with an eosinophilic inflammation, asthma after bronchiolitis is less related to atopy and mainly associated with viral induced wheeze and bronchial inflammation mediated by neutrophils [12-14]. Thus, markers of inflammation such as FeNO could conceivably be different in asthma after bronchiolitis than in children with atopic asthma.

The primary aim of this study was to assess if FeNO was different in children with former hospitalization for bronchiolitis compared to a control group, and secondly to explore whether the role of FeNO as a marker of asthma, atopy or BHR differed between these two groups of children.

## Methods

### Study design and subjects

In this longitudinal prospective follow-up study, children below 12 months of age hospitalized for acute bronchiolitis at the university hospitals in Stavanger and Bergen (Norway) during the winter seasons 1997 and 1998 were invited to participate. Bronchiolitis was defined as an acute febrile episode of respiratory illness with tachypnea, dyspnoea, prolonged expiration and wheeze on auscultation of the chest. Exclusion criteria were previous hospitalization for wheeze or bronchiolitis, any previous use of systemic or inhaled corticosteroids, signs of bacterial infection or any other known lung disease [15]. Nasopharyngeal mucus was examined for Respiratory syncytial virus (RSV) by direct immunofluorescence in all patients (bioMèrieux, Marcy-l’Ètoile, France). Other viruses were not systematically tested for.

The children were invited to a follow-up in 2008–09 at 11 years of age. The follow-up included a questionnaire from the International Study of Asthma and Allergy in Childhood (ISAAC) [16], assessment of lung function and BHR, FeNO measurement and skin prick tests (SPT). An unselected age matched control group not hospitalized for bronchiolitis during their first year of life, reflecting the general population in the study area was recruited from 3 nearby schools.

We have previously published results showing that children in the post-bronchiolitis group had more asthma, lower lung function and higher BHR compared to controls [10].

The study was approved by the Regional Committee for Medical and Health Research Ethics West, and signed statements of informed consent were obtained from all parents.

### Lung function measurements

Spirometry was performed according to established guidelines [17], using a Vmax Encore 229D spirometer (SensorMedics Inc., Anaheim, USA). Forced expiratory volume in first second (FEV_1_), forced vital capacity (FVC) and forced expiratory flows at 25-75% of FVC (FEF_25-75_) were recorded. Except for the ratio FEV_1_/FVC, measurements were compared to values predicted by standard reference equations and expressed as percentages of predicted FEV_1_% [18] and FEF_25-75_% [19]. BHR was assessed with methacholine provocation test (MPT), by using an inhalation-synchronised, dosimetric nebulizer, Spira Elektra 2® (Spira, Hämeenlinna, Finland). The test was not performed if baseline FEV_1_% was <65% predicted. Methacholine was administered in doubling doses until a 20% reduction in FEV_1_ was obtained or until a cumulative dose of 11.54 *μ*mol had been given. A dose response slope (DRS) was calculated as the ratio between the maximum percentage decline in FEV_1_ from baseline and the total administered dose of methacholine (%/*μ*mol), and the distribution regarded as ln-normal [20].

### FeNO measurements

FeNO was measured online by the single breath technique according to published guidelines [21], with an EcoMedics Exhalyzer ® CLD 88sp with DENOX 88 (ECO MEDICS AG, Duernten, Switzerland). NO-free air was inhaled to near total lung capacity, followed immediately by full exhalation at a constant flow of 50 ml/s. FeNO was recorded as the mean value from 3 reproducible plateaus within 10% acceptability.

### Skin prick tests

Skin prick tests (SPT) with the most common inhalant allergens (*Dermatophagoides pteronyssinus*, dog, cat dander, *Cladosporium herbarium*, birch, timothy, German cockroach) and food allergens (eggwhite, milk, peanut, codfish) (Soluprick®, ALK Albello, Hørsholm, Denmark) for atopic sensitization in Norwegian children were performed [22]. Histamine 10 mg/ml was used as a positive control and a 0.9% saline solution as a negative control. A wheal diameter ≥ 3 mm larger than the negative control was defined as a positive result.

### Definitions

Current asthma at 11 years of age was defined as a positive answer to the ISAAC question regarding “asthma ever” and a positive answer to at least one of the two questions:

^1)^ wheezing or whistling in the chest or chest tightness during the preceding 12 months or ^2)^ use of asthma medication (bronchodilators, inhaled corticosteroid, leukotriene antagonists) during the preceding 12 months.

The children in the post-bronchiolitis and control groups were divided into four sub-groups at the 11 year follow-up, according to their atopic and asthmatic status. ^1)^ Healthy: No current asthma and no allergic sensitization. ^2)^ Atopic non-asthmatic: Positive SPT for at least one allergen with the absence of current asthma. ^3)^ Current atopic asthma: A combination of current asthma and atopy. ^4)^ Current non-atopic asthma: Current asthma without atopy.

### Statistical methods

Means and standard deviations (SD), medians and quartiles were estimated and reported for normally and asymmetrically distributed data, as appropriate. Group comparisons were done with Student’s *t*- test, Mann Whitney *U-*test or Pearson’s chi-square exact test, as appropriate. FeNO (unit: parts per billion (ppb)) was regarded as ln-normally distributed and results presented as back-transformed values given as geometric means with 95% confidence intervals (CI). To study overall associations with FeNO, a two-way ANOVA was performed for the post-bronchiolitis and control groups in one common analysis. To study associations with FeNO for each sub-group, the post-bronchiolitis and control groups were analysed separately and Dunnett’s test was used for post-hoc comparisons between the sub-groups if the F-test was significant in the overall ANOVA analysis.

Linear regression analyses were applied to explore associations between putative explanatory variables and ln FeNO for the complete study group and for the post-bronchiolitis group separately. In both models, the following variables recorded at 11 years of age were assessed: Gender, age at follow-up, height, weight, atopy, current asthma, ln DRS, FEV_1_%, FEF_25–75_%, use of inhaled steroids the preceding 12 months and previous hospitalization for bronchiolitis in infancy. In the separate multivariate linear regression analysis including only subjects in the post-bronchiolitis group, RSV status (positive or negative) was also included in addition to those included for the complete study group. In all analyses, each variable was initially entered into a univariate model. Variables with p-values < 0.2 in univariate analyses were further analysed in a backward multivariate regression model. Analyses of interaction terms were used to explore differences between the sub-groups regarding associations between explanatory variables and FeNO. When ln transforming DRS, negative values were set to 0.001. P-values < 0.05 were regarded as statistically significant. All analyses were two-tailed and data were analyzed using the SPSS version 18.0 statistical package (SPSS, Chicago, IL, USA).

## Results

One hundred and thirty one children hospitalized for bronchiolitis during their first year of life were included, and 108 (82%) consented to the follow-up at 11 years of age. Of these, 82 children (76%) had tested positive for RSV. All completed the questionnaire and took part in SPT and lung function tests. MPT and FeNO were not performed in two and three children in the post-bronchiolitis group respectively, due to technical reasons.

In the control group, 91 of the 190 primarily invited children (48%) completed the questionnaire and agreed to SPT and lung function test; one was excluded as further investigations indicated chronic restrictive lung disease. One child was not able to perform neither spirometry, FeNO nor MPT. In addition, MPT was not performed in two children; one had FEV_1_% < 65% and one was not able to cooperate.

In the post-bronchiolitis group, FEV_1_% was lower in the healthy group compared to the atopic non-asthmatic group (Tables 1 and 2). There were no other differences between the four sub-groups regarding gender, age, weight, height, lung function and BHR at the 11 year follow-up within the post-bronchiolitis group and the control group, respectively (Tables 1 and 2).

**Table 1:** Characteristics of 108 children hospitalized for bronchiolitis in their first year of life during 1997–98 at the university hospitals of Stavanger and Bergen (Norway) according to asthma and atopy at 11 years of age

	**Healthy (n = 64)**	**Atopic non-asthmatic (n = 20)**	**P-value**	**Current non-atopic asthma (n = 15)**	**P-value**	**Current atopic asthma (n = 9)**	**P-value**
Boys, n (% of group)	30 (47)	14 (70)	0.080	10 (67)	0.252	6 (67)	0.308
Age at hospitalization^*^ (months)	3.5 (2.0, 6.0)	4.0 (1.0, 10.0)	0.603	6.0 (3.0, 9.0)	0.052	5.0 (4.0, 9.5)	0.083
Age at follow up^*^ (year)	11.4 (11.0, 11.8)	11.3 (10.9, 11.5)	0.182	11.4 (10.9, 11.6)	0.745	11.6 (11.3, 12.2)	0.410
Weight at follow-up^†^ (kg)	41.2 (9.3)	40.6 (8.2)	0.791	42.8 (7.5)	0.525	42.5 (8.3)	0.692
Height at follow-up^†^ (cm)	149.0 (8.2)	147.2 (4.3)	0.206	149.3 (5.6)	0.888	148.9 (6.0)	0.963
ICS, n (% of group)	0	0		2 (13)	**0.034**	7 (78)	**<0.001**
FEV_1_%^†^	93.9 (9.3)	99.7 (8.9)	**0.014**	97.8 (9.5)	0.145	95.8 (9.5)	0.555
FEF_25–75_%^†^	89.9 (23.9)	88.6 (19.1)	0.819	86.0 (22.9)	0.568	84.4 (20.2)	0.514
FEV_1_ /FVC ratio^†^	82.8 (7.5)	80.6 (4.9)	0.221	79.7 (7.7)	0.159	78.8 (7.3)	0.135
DRS to methacholine^*^	6.0 (1.7, 25.5)	4.9 (1.1, 13.4)	0.378	4.1 (1.5, 18.1)	0.910	4.4 (2.2, 13.8)	0.923

^*^Median (inter quartile range), ^†^mean (standard deviation). ICS, inhalation corticosteroid last 12 months before follow up; FEV_1_%, forced expiratory volume in first second as percentage of predicted; FEF_25–75_%, forced expiratory flow between 25-75% of the forced vital capacity (FVC); DRS, dose response slope. For missing data, see text. P-values assess comparisons with the healthy group.

Bold values indicate significance at the 0.05 level.

**Table 2:** Characteristics of 90 children in an age matched control group at 11 years of age, according to asthma and atopy

	**Healthy (n = 51)**	**Atopic non-asthmatic (n = 29)**	**P-value**	**Current non-atopic asthma (n = 5)**	**P-value**	**Current atopic asthma (n = 5)**	**P-value**
Boys, n (%)	31 (61)	16 (55)	0.644	4 (80)	0.640	4 (80)	0.640
Age at follow up^*^ (year)	11.8 (11.4, 12.2)	11.4 (11.0, 12.1)	0.081	12.3 (11.4, 12.8)	0.147	11.8 (10.9, 12.0)	0.502
Weight at follow-up^†^ (kg)	41.7 (8.5)	40.9 (9.4)	0.677	47.9 (17.3)	0.472	51.4 (25.7)	0.449
Height at follow-up^†^ (cm)	151.9 (7.6)	149.0 (7.0)	0.087	150.8 (10.2)	0.749	152.0 (17.1)	0.998
ICS, n (%)	1 (2)	0	1.000	3 (60)	**0.001**	4 (80)	**<0.001**
FEV_1_%^†^	98.7 (10.6)	99.8 (7.9)	0.631	101.9 (10.7)	0.510	96.3 (28.3)	0.864
FEF_25–75_%^†^	96.9 (22.9)	98.9 (16.1)	0.693	95.6 (18.9)	0.903	93.3 (45.7)	0.764
FEV_1_ /FVC ratio^†^	84.3 (6.6)	84.2 (4.5)	0.969	79.8 (4.2)	0.146	80.8 (6.7)	0.269
DRS to methacholine^*^	1.7 (1.0, 6.8)	3.6 (0.8,17.6)	0.084	0.9 (0.3, 5.8)	0.292	4.7 (1.1, 24.5)	0.405

^*^Median (inter quartile range), ^†^mean (standard deviation). ICS, inhaled corticosteroids last 12 months before follow up; FEV_1_%, forced expiratory volume in first second as percentage of predicted; FEF_25–75_%, forced expiratory flow between 25-75% of the forced vital capacity (FVC) as percentage of predicted; DRS, dose response slope. For missing data, see text. P-values assess comparisons with the healthy group.

Bold values indicate significance at the 0.05 level.

Children in the post-bronchiolitis group (11.4 years; 11.0, 11.7) (median; quartiles) were slightly younger than the controls (11.7 years; 11.3, 12.1) at the 11 year follow-up (p < 0.001).

### FeNO

**Figure 1:**
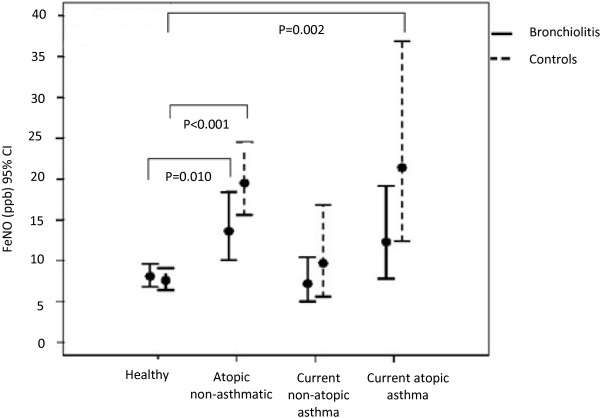
**FeNO levels in four different sub-groups of children, split by bronchiolitis status in their first year of life.** FeNO values are given as geometric mean with 95% confidence intervals (CI).

The overall ANOVA analysis with all children included, revealed that FeNO levels did not differ between the post-bronchiolitis and control groups (p = 0.214) (Table 3). FeNO differed between the four sub-groups (p < 0.001). FeNO levels were higher in the atopic non-asthmatic and the atopic asthmatic children but not in the children with non-atopic asthma compared to healthy in both the post-bronchiolitis group and in the control group (Table 3). Separate analyses for the post-bronchiolitis and the control group revealed that FeNO was higher in the atopic non-asthmatic children compared to healthy in both groups. Higher FeNO in children with atopic asthma compared to healthy was observed only in the control group (Table 4, Figure 1).

**Table 3:** Analysis of variance for fractional exhaled nitric oxide (FeNO) given as ln FeNO in children hospitalized for bronchiolitis (n = 105) during their first year of life and an age matched control group (n = 89) at 11 years of age

**Variable**	B^*^	**95% CI**	**P-value**^**†**^
**Main groups**			
Control group	0	Reference	
Post-bronchiolitis group	−0.120	−0.309, 0.070	0.214
**Sub-groups by atopy and asthma status**			**<0.001**
Healthy	0	Reference	
Atopic non-asthmatic	0.745	0.522, 0.967	
Current non-atopic asthma	0.013	−0.308, 0.335	
Current atopic asthma	0.651	0.286, 1.102	
Intercept^‡^	2.131	1.970, 2.291	

No significant interaction effects were observed between the variables post-bronchiolitis/control group and the four subgroups of the study, i.e. the relationships between FeNO values measured in these four subgroups were similar in the post-bronchiolitis and the control group.

^*^Regression coefficient; represents the amount of change of ln NO induced by a change of 1 unit of the explanatory variable.

^†^P-values from F test. ^‡^Reference group (healthy children in the control group).

Bold values indicate significance at the 0.05 level.

**Table 4:** Levels of fractional exhaled nitric oxide (FeNO) in children hospitalized for bronchiolitis and in an age matched control group, by asthma and atopic status

		**Post-bronchiolitis group (n = 105)**			**Control group (n = 89)**			
	**N**	**FeNO**	**95% CI**	P-values^* ^**vs. healthy**	**N**	**FeNO**	**95% CI**	P-values^*^vs. healthy
Healthy	62	8.1	6.8, 9.6	Reference	50	7.6	6.4, 9.1	Reference
Atopic non-asthmatic	20	13.6	10.1, 18.4	**0.010**	29	19.5	15.6, 24.5	**<0.001**
Current non-atopic asthma	14	7.2	5.0, 10.4	0.920	5	9.7	5.6, 16.8	0.781
Current atopic asthma	9	12.3	7.8, 19.2	0.237	5	21.4	12.4, 36.9	**0.002**

Figures are geometric means and 95% confidence intervals (95% CI). FeNO values are given as parts per billion. ^*^Dunnett’s test.

Bold values indicate significance at the 0.05 level.

### Regression analyses of potential explanatory factors for ln FeNO

Atopy, weight and height were positively associated with ln FeNO by univariate linear regression analyses including all participating children (Table 5). In the final multivariate model, atopy, ln DRS and height were independently associated with increased ln FeNO (Table 5). No interaction effects regarding ln FeNO were observed between the variables atopy and current asthma vs. no asthma, meaning that the associations between atopy and ln FeNO were similar for the asthmatic and non-asthmatic children and vice versa (Table 5).

**Table 5:** Linear regression model explaining fractional exhaled nitric oxide (FeNO) given as ln FeNO at 11 years of age in 105 children hospitalized for bronchiolitis and 89 children in an age matched control group, all children analysed together

	**Unadjusted models**			**Fully adjusted model (N = 190)**			**Final model (N = 190)**		
**Risk factors**	B^*^	**95% CI**	**P-value**	B^*^	**95% CI**	**P-value**	B^*^	**95% CI**	**P-value**
Hospitalization for bronchiolitis	−0.200	−0.408, 0.008	0.059	−0.088	−0.276, 0.101	0.359			
Male gender	−0.131	−0.342, 0.080	0.223						
Age at follow up (months)	0.009	−0.006, 0.024	0.257						
Height (cm)	0.021	0.007, 0.034	**0.002**	0.025	0.008, 0.042	**0.005**	0.027	0.015, 0.038	**<0.001**
Weight (kg)	0.014	0.004, 0.025	**0.009**	0.001	−0.012, 0.014	0.849			
Atopy	0.736	0.539, 0.934	**<0.001**	0.757	0.562, 0.951	**<0.001**	0.773	0.583, 0.962	**<0.001**
Current asthma	0.035	−0.243, 0.313	0.805						
Ln DRS	0.053	−0.003, 0.108	0.062	0.056	0.008, 0.105	**0.023**	0.051	0.004, 0.097	**0.034**
FEV_1_%	0.006	−0.004, 0.016	0.269						
FEF_25-75_%	0.001	−0.003, 0.006	0.597						
Use of inhaled steroids preceding 12 months	0.200	−0.169, 0.568	0.287						

No interactions were found between current asthma and atopy, atopy and DRS, atopy and hospitalization for bronchiolitis or DRS and hospitalization for bronchiolitis.

^*^Regression coefficient, represents the amount of change of ln NO induced by a change of 1 unit of the explanatory variable.

CI, confidence interval; DRS, dose response slope; FEV_1_%, forced expiratory volume in first second as percentage of predicted; FEF_25-75_%, forced expiratory flow between 25-75% of the forced vital capacity.

Bold values indicate significance at the 0.05 level.

Separate regression analyses were done for children in the post-bronchiolitis group. By univariate analyses, RSV negative bronchiolitis, height, FEV_1_% predicted and atopy were positively associated with ln FeNO (Table 6). In the final multivariate model, ln DRS, height and FEV_1_% predicted were positively associated with ln FeNO (Table 6). There was a significant interaction effect between RSV negative bronchiolitis and atopy, i.e. atopy was positively associated with FeNO in the RSV negative group (B = 1.005; 95% CI: 0.496, 1.513; p < 0.001), but not in the RSV positive group (B = 0.269; 95% CI: -0.071, 0.609; p = 0.120) Table 6.

**Table 6:** Linear regression model explaining fractional exhaled nitric oxide (FeNO) given as ln FeNO at 11 years of age in 105 children hospitalized for bronchiolitis

	**Unadjusted models**			**Fully adjusted model (N = 103)**			**Final model (N = 103)**		
**Risk factors**	B^*^	**95% CI**	**P-value**	B^*^	**95% CI**	**P-value**	B^*^	**95% CI**	**P- value**
RSV negative bronchiolitis	0.335	0.019, 0.651	**0.038**	0.010	−0.337, 0.357	0.957	0.009	−0.336, 0.354	0.958
Male gender	−0.112	−0.388, 0.164	0.423						
Age at follow-up (months)	−0.004	−0.277, 0.269	0.977						
Height (cm)	0.024	0.005, 0.043	**0.015**	0.025	0.000, 0.050	0.053	0.024	0.007, 0.041	**0.006**
Weight (kg)	0.015	0.001, 0.030	0.058	−0.001	−0.021, 0.020	0.946			
Atopy	0.510	0.219, 0.802	**0.001**	0.269	−0.073, 0.611	0.121	0.269	−0.071, 0.609	0.120
Current asthma	−0.033	−0.366 ,0.299	0.977						
Ln DRS	0.061	−0.025, 0.148	0.161	0.094	0.015, 0.173	**0.020**	0.094	0.017, 0.172	**0.018**
FEV_1_%	0.015	0.000, 0.029	**0.044**	0.018	0.004, 0.032	**0.013**	0.018	0.004, 0.031	**0.012**
FEF_25–75_%	0.002	−0.005, 0.008	0.610						
Use of inhaled steroids preceding 12 months	0.003	−0.494, 0.488	0.990						
**Interaction**									
RSV negative bronchiolitis × atopy				0.736	0.125, 1.346	**0.019**	0.736	0.128, 1.343	**0.018**

No interactions were found between current asthma and atopy, atopy and ln DRS or ln DRS and RSV negative bronchiolitis. There was an interaction between RSV negative bronchiolitis and atopy, and the final model therefor includes both the interaction effect and its main variables (atopy and RSV negative bronchiolitis).

^*^Regression coefficient, represents the amount of change of ln NO induced by a change of 1 unit of the explanatory variable.

CI, confidence interval; DRS, dose response slope; FEV_1_%, forced expiratory volume in first second as percentage of predicted; FEF_25–75_%, forced expiratory flow between 25-75% of the forced vital capacity.

Bold values indicate significance at the 0.05 level.

There was no significant association between RSV negative bronchiolitis and atopy (Pearson’s chi square exact test p =0.304), and there was no association between atopy and ln DRS (B = 0.173; 95% CI: -0.409, 0.755; p =0.558).

## Discussion

In the present study FeNO did not differ between 11 year old children hospitalized for bronchiolitis in their first year of life and an age matched control group. Secondly, atopy and BHR, but not asthma were associated with FeNO, and these associations were similar in the post-bronchiolitis and in the control groups.

The guideline from the American Thoracic Society suggests that levels of FeNO below 20 ppb are less likely to indicate eosinophilic airway inflammation [2], as also reported by others [8]. In the present study, the majority of the FeNO measurements were below this limit. Infantile wheeze has been associated mainly with a neutrophilic inflammation, and a tendency for continued neutrophilic inflammation in this group of children could conceivably contribute to the findings of the present study [12].

The association between FeNO and atopy, but not between FeNO and asthma is in line with several other studies [3-5]. An association between FeNO and persistent wheezing has been reported for children less than 2–3 years of age [23,24], but we could not confirm that this association lasts until adolescence. One of these studies did not adjust for atopy [23], while another study observed that neither personal nor a family history of atopy was associated with increased FeNO [24]. Konstantinou et al. recently described an episodic increase of FeNO during viral wheezing in 4–6 year old children, independent of the atopic status of the test-subjects. The increase subsided after the episodes resolved, rendering wheezers comparable to non-wheezers outside the wheezing episodes [25]. This is consistent with the low levels of FeNO in the post-bronchiolitis group in the present study. Others have reported associations between FeNO and recurrent wheeze in infants with an atopic constitution [26] and in atopic children younger than four years of age [27]. The results from the present study suggest that also for older children with a history of infant and preschool viral wheeze, atopy should be considered as an independent risk factor for increased FeNO_._ This is in line with a study from the Netherlands showing that FeNO can differentiate between wheezing phenotypes, but only in atopic children [28].

As previously published, lower FEF_25–75_% predicted, consistent with small airway obstruction, was observed in children in the post-bronchiolitis group than children in the control group [10]. Except for a weak positive association between FEV_1_% and FeNO in the post-bronchiolitis group, no associations between lung function and FeNO could be observed. FeNO may predict lung function decline in adults with severe asthma [29]. However, to our knowledge, few studies have found associations between lung function and FeNO in children [30]. Low levels of FeNO despite small airway obstruction could indicate structural explanatory mechanisms and not an ongoing eosinophilic inflammation [31].

### RSV positive vs. negative bronchiolitis

Asthma after bronchiolitis in infancy is heterogeneous and likely to represent disease entities that differ from atopic asthma in childhood. RSV is the most common virus involved in bronchiolitis, but apart from one Swedish study [32], the risk of asthma after RSV bronchiolitis has not been linked to atopy [9,11]. An increased risk of asthma after RSV negative vs. RSV positive bronchiolitis has been reported [10], particularly after RV bronchiolitis [11]. Wheezing with RV infections has been associated with atopy [9,33], although we found no association between atopy and a history of RSV negative bronchiolitis. Temporarily reduced FeNO has been found in children hospitalized for RSV bronchiolitis. Although the explanatory mechanisms are not known, it has been speculated if the absence of eosinophilic inflammation during the acute bronchiolitis may be involved [34]. The present study showed that previous RSV negative bronchiolitis was associated with higher FeNO, but not after adjusting for DRS, atopy and height.

The interaction effect observed between atopy and RSV negative bronchiolitis may suggest that the influence from atopy on FeNO is different for children with former RSV positive than RSV negative bronchiolitis. Our results could indicate that atopy was more linked to FeNO at 11 years of age in children with former RSV negative than RSV positive bronchiolitis. However, the number of participants was limited and there was a similar and near significant tendency also for children with a history of RSV positive bronchiolitis, suggesting that the results should be interpreted with caution.

### FeNO and bronchial hyperresponsiveness

In the present study, DRS to methacholine was independently and positively associated with FeNO by the multivariate regression analyses including all children and also in the separate regression analyses including only children in the post-bronchiolitis group.

A similar association was found by Franklin et al., but only in atopic children [5]. The Copenhagen birth cohort study observed an association between FeNO and BHR, but underlined that this association was independent of asthma symptoms [35]. We found a similar association between DRS and FeNO for atopic and non-atopic children. In the present and in another recent study from the same population, asthma or atopy was not associated with BHR, although BHR was higher in the post-bronchiolitis group [10]. The relationship between NO metabolism and BHR in asthma is complex [36]. The ATS guideline underlines that studies report inconsistent associations and low correlations between FeNO and BHR, and that BHR, airway inflammation and FeNO belong to different domains [2]. A Norwegian twin study observed that common genetic effects could explain the association between FeNO and BHR, suggesting that FeNO is not related to BHR per se [37]. Moreover, BHR measured by direct provocation tests using methacholine or histamine reflects structural airway changes, compared to indirect provocation tests such as adenosine monophosphate or exercise which probably better reflects airway inflammation [38].

### Strengths and limitations

The main strengths of this study were the prospective design, the long follow-up period and the high attendance rate of 82% of those originally included with bronchiolitis. However, the number of participants in the various sub-groups was relatively low, reducing the statistical power and complicating the interpretations of the results. This could impact the lack of interaction between the subgroups and between the post-bronchiolitis and control groups in the overall ANOVA analysis. In addition, the number of children in the RSV negative group was small and the results regarding this group must be interpreted with caution.

The children in the control group were slightly older than children in the post-bronchiolitis group at follow-up, but this should not influence the predicted values regarding lung function. However, a selection bias among those who consented cannot be excluded.

RSV was analyzed by direct immunofluorescence and not based on nucleic acid amplification such as reverse polymerase chain reaction (PCR). PCR is considered more sensitive and specific than direct immunofluorescence [39].

## Conclusion

In this study, FeNO did not differ between 11 year old children hospitalized for bronchiolitis in infancy and an age matched control group. FeNO was associated with atopy, but not with asthma in both groups of children. This may suggest that FeNO may be unrelated to the pathophysiology of asthma after bronchiolitis. The results could also reflect that airway inflammation is rare in children 11 years after bronchiolitis.

## Abbreviations

BHR: Bronchial hyperresponsiveness; FeNO: Exhaled nitric oxide; RSV: Respiratory syncytial virus; ISAAC: International Study of Asthma and Allergy in Childhood; SPT: Skin prick test; MPT: Methacholine provocation test; ICS: Inhaled corticosteroid; FEV1%: Forced expiratory volume in first second as percentage of predicted; FEF25–75%: Forced expiratory flow between 25-75% of the forced vital capacity; FVC: Forced vital capacity as percentage of predicted; DRS: Dose response slope; PCR: Polymerase chain reaction.

## Competing interests

The authors declare that they have no competing interests.

## Authors’ contributions

IBM: Participated in drafting the study, performed the sampling of data at Stavanger University Hospital, performed the statistical analyses, wrote a draft and completed the manuscript. TH: Contributed to the draft of the study, was responsible for the sampling of data at Haukeland University Hospital, and contributed significantly to the writing of the manuscript. KØ: Supervised all parts of the study, the drafting, registration of data, analyses and contributed significantly to the writing of the manuscript. All authors read and approved the final manuscript.

## Pre-publication history

The pre-publication history for this paper can be accessed here:

http://www.biomedcentral.com/1471-2466/13/66/prepub
